# Enhancing interpretability for Bayesian basket trial designs by effective sample size

**DOI:** 10.1186/s12874-025-02715-x

**Published:** 2025-12-16

**Authors:** Xin Chen, Jingyi Zhang, Wenyun Yang, Liyun Jiang, Bosheng Li, Fangrong Yan

**Affiliations:** Department of Biostatistics, China Pharmaceutical University, Nanjing, China

**Keywords:** Basket trial, Bayesian clinical trial design, Information borrowing, Effective sample size, Bayesian hierarchical model

## Abstract

**Background:**

There is growing interest in utilizing Bayesian approaches to borrow information across tumor types in basket trials. Several innovative designs, primarily extensions of the Bayesian hierarchical model (BHM), have been proposed to dynamically borrow information based on observed data. However, there is no recognized solution to quantify the degree of information borrowing in such a context, posing a great challenge for non-statisticians to understand these complex designs.

**Methods:**

The tool of effective sample size (ESS) is leveraged to Bayesian basket trials and several ESS-based borrowing strategies are proposed. The mean squared error (MSE), which explicitly accounts for the trade-off between estimation bias and variance reduction, is selected as the target measure for deriving ESS. Through a reanalysis of the RAGNAR study as well as simulation studies, the interpretability of ESS is demonstrated at both the analysis and design stages of basket trials.

**Results:**

ESS reflects the impact of information borrowing on MSE and intuitively characterizes the degree of borrowing. It aligns with the type I error rate and power, showing potential as a valuable complement in statistical analyses and simulation studies.

**Conclusions:**

Quantifying the degree of information borrowing by ESS can greatly help trialists design Bayesian basket trials, reasonably evaluate and interpret the results of Bayesian analyses, conduct sensitivity analyses, and ultimately borrow proper amount of information in basket trials.

**Supplementary Information:**

The online version contains supplementary material available at 10.1186/s12874-025-02715-x.

## Background

With the advancement of precision oncology and the advent of targeted therapies, the landscape of clinical trials has undergone a significant transformation. The basket trial, a type of master protocol, is designed to simultaneously test an investigational therapy across multiple tumor types (or indications) that share common molecular alterations [1]. This tumor-agnostic fashion not only expands access to novel treatments for rare cancer populations but also improves trial efficiency by utilizing a shared clinical research infrastructure for multiple sub-studies (each sub-study corresponding to a specific indication). To date, several tumor-agnostic therapies have been approved by the US Food and Drug Administration (FDA), and the readers may refer to recent reviews for summaries of regulatory approvals and the evolution of basket trials in the field of cancer drug development [2–5].

Regarding the primary statistical analysis of basket trials, pooled analysis among indications and independent indication-specific analysis are the two most commonly used approaches [6], though there are several limitations. The pooled analysis assumes a consistent treatment effect across all indications, which may not be valid in many cases. One typical example is the basket trial of vemurafenib in multiple nonmelanoma cancers with BRAF V600 mutations, where the overall response rate (ORR) was 42% for the cohort of patients with non–small-cell lung cancer (NSCLC) but was almost 0 for colorectal cancer [7]. Conversely, the independent analysis often suffers from low statistical power, particularly for rare cancers with limited sample sizes. Consequently, there has been growing interest in Bayesian basket trial designs, which enable information sharing across indications to address these challenges.

Bayesian basket trial designs, primarily exemplified by the Bayesian hierarchical model (BHM) and its extensions, were mostly proposed to dynamically borrow information across indications [8–13], representing a middle ground between pooled analysis (assuming complete homogeneity across indications) and independent analysis (assuming complete heterogeneity) [14]. The BHM has been applied in various basket trials to support decision-making [15–21]. However, if the investigational therapy demonstrates efficacy in only a subset of indications, information borrowing may introduce risks of bias and type I error inflation. To mitigate these risks, it is essential to carefully evaluate the rationale of information borrowing before implementing Bayesian approaches. Key considerations include the similarity of pathological features, the mechanism of action (MOA) of the therapy, and the alignment of primary outcome measures across indications, etc [22]. From these clinical perspectives, investigators and research teams often have prior beliefs on appropriate extent of information borrowing. Regulatory agencies may also impose constraints on the degree of information borrowing permitted [23, 24]. For any given indication, it is crucial to avoid cases where borrowed information dominates statistical inferences, potentially obscuring evidence from that indication itself. Conversely, borrowing too little may diminish the advantages of Bayesian approaches. Therefore, achieving an acceptable level of borrowing is critical to maintaining both the validity and efficiency of Bayesian basket trials.

To the best of our knowledge, existing methods for quantifying the degree of borrowing in basket trials lack sufficient intuitiveness, e.g., posterior estimate of the between-group variance [25, 26]. While a smaller variance indicates stronger borrowing, the practical interpretation of specific variance values remains challenging. Morita et al. (2012) proposed quantifying the degree of borrowing using the concept of effective sample size (ESS), where a specific prior ESS can be determined for each indication upon pre-specifying the BHM [27]. This approach provides a more interpretable metric compared to the between-group variance. However, this type of prior ESS, as well as those based on variance ratios [28–30], are not always ideal for decision-making, as they do not account for estimation bias of the treatment effect. Specifically, these methods emphasize the reduction of posterior variance while ignoring potential bias introduced by Bayesian modeling frameworks. As a basket trial is usually an exploratory, phase II proof-of-concept (POC) study [31, 32], the primary objective is to figure out whether the investigational therapy could provide desired treatment effect and merit further pursuit. If the estimated treatment effects are promising for some indications, the sponsors may continue the clinical developments (i.e., making Go decisions). Conversely, if no efficacy is observed, the sponsors should make No-Go decisions. This decision-making process actually depends on the estimates of treatment effects rather than a formal hypothesis testing, although type I error rate and power are often important metrics in calibrating design parameters for Bayesian basket trials. But in a POC study, a binary Go/No-Go decision based on the primary endpoint just provides a reference, and strict type I error control is not always enforced. For example, if the *p*-value for testing a pre-specified null hypothesis is 0.051, will the clinical development for that indication be stopped? It is expected that most sponsors will examine trial data to see if there are any other benefits for patients, instead of announcing a halt to drug development. In fact, making Go/No-Go decisions is a multi-faceted consideration based on primary and secondary efficacy endpoints, concordance between primary and secondary endpoints, safety profiles, etc. The real benefit of applying Bayesian approaches to borrow information is to obtain a better balance of the bias-variance tradeoff, i.e., reducing the uncertainty of estimation in scenarios where the treatment effects are similar across indications, at the cost of certain increased risk of bias in scenarios with high heterogeneity [22]. The mean squared error (MSE), defined as the average of error squares between the estimated and true values, is a natural measure of bias-variance tradeoff. In several publications regarding basket trial designs, the MSE is also regarded as an important metric in the simulation studies [10, 12, 13]. Therefore, we adopt the MSE-based ESS framework [32, 33] to enhance the interpretability of information borrowing in Bayesian basket trials. This approach explicitly addresses prior-data conflicts (at the analysis stage) and prior-likelihood conflicts (at the design stage) by accounting for the bias-variance tradeoff inherent in borrowing. It advances beyond conventional ESS definitions by penalizing conditions where borrowing introduces substantial bias. This is critical in basket trials, where borrowing heterogeneous information across indications may bias treatment effect estimates and even lead to erroneous Go/No-Go decisions.

## Methods

### Prior effective sample size (ESS)

Before introducing our proposal, it is necessary to first outline the basic framework of prior ESS [34–37]. Let $\pi\left(\theta\right)$ denote the target prior distribution for parameter(s) $\theta$, and there is a vague prior ${\pi}_{0}\left(\theta\right)$. The posterior distribution $\pi\left(\theta|{Y}_{n}\right)$ is composed of $\pi\left(\theta\right)$ and information from a sample of size $n$. Similarly, posterior distribution ${\pi}_{0}\left(\theta|{Y}_{m+n}\right)$ is derived by updating ${\pi}_{0}\left(\theta\right)$ with a hypothetical sample of size $\:m+n$. The basic idea for determining the prior ESS of $\pi\left(\theta\right)$ is matching $\pi\left(\theta|{Y}_{n}\right)$ and ${\pi}_{0}\left(\theta|{Y}_{m+n}\right)$, i.e., minimizing a certain distance as follows:

1 $$\begin{array}{c}ESS=\text{arg}\underset m{\text{min}}\left|D\left\{{\pi}_0\left(\theta\vert Y_{m+n}\right),\pi\left(\theta\vert Y_n\right)\right\}\right|\end{array}$$

where $\:D\{\cdot\}$ is a user-defined target measure for quantifying the distance, such as the relative entropy (Kullback-Leibler divergence), difference in variance, curvature of log-density, or MSE. If such $\:m$ can be solved, it is intuitive to conclude the prior ESS of $\pi\left(\theta\right)$ is $\:m$, as information of ${Y}_{m+n}$ is equal to the information of ${Y}_{n}$ plus $\pi\left(\theta\right)$ (approximately or exactly). When $n=0$, (1) degenerates to matching $\pi\left(\theta\right)$ with ${\pi}_{0}\left(\theta|{Y}_{m}\right)$, corresponding to the foundational work of Clarke (1996) [37] and Morita et al. (2008) [36]. When $n>0$, it means the current trial design and/or data is taken into account. In the context of basket trials, considering the case $\:n>0$ is particularly critical as data from other indications would influence the degree of borrowing. 

For conjugate priors, the ESS is well established, where a conjugate prior $\pi\left(\theta\right)$ can be interpreted as arising from a vague prior ${\pi}_{0}\left(\theta\right)$ of the same distribution family, augmented by a dataset of *ESS* observations. For example, consider a Binomial random variable $Y_n\sim Bin\left(n,\mathrm\theta\right)$ with a size of $n$ and success probability $\theta$. The conjugate prior $Beta(a,b)$ for $\theta$ corresponds to an ESS of $\:a+b$. Essentially, this is equivalent to matching $\pi\left(\theta\right)$ and ${\pi}_{0}\left(\theta\right|{y}_{m})$, where ${\pi}_{0}\left(\theta\right)$ is $Beta({a}_{0},{b}_{0})$ ($\:{a}_{0}$ and $\:{b}_{0}$ are arbitrarily small values) and $\:{y}_{m}$ is a hypothetical sample with $a-{a}_{0}$ successes and $\:b-{b}_{0}$ failures. Then, the prior ESS derived by distribution matching equals $a-{a}_{0}+b-{b}_{0}\approx\:a+b$. 

In terms of nonconjugate priors, ESS quantification becomes non-trivial and usually requires matching distributions through Eq. (1). The readers may refer to Neuenschwander et al. (2020) [38] and Reimherr et al. (2021) [34] for comprehensive reviews of ESS.

### Determining ESS in Bayesian basket trials

Based on the ESS methodology introduced earlier, this section describes how to determine ESS in Bayesian basket trials. It is important to highlight that in these trials, data are collected simultaneously across all indications, and information borrowed from other indications is not temporal “prior information” in the conventional sense. Within this paper, we expand the term “prior ESS” to quantify such borrowed information for a specific indication.

Consider a single-arm basket trial evaluating an investigational therapy for $\:K$ indications. Let ${Y}_{k}(k=1,\dots,K)$ denote the number of responders among $\:{n}_{k}$ patients for indication $k$, with ${p}_{k}$ representing the true response probability. A typical BHM is specified as:

$$Y_k\sim\mathrm{Bin}\left(n_k,p_k\right)$$

$${\theta}_{k}=\text{l}\text{o}\text{g}\text{i}\text{t}\left({p}_{k}\right)$$

2 $$\begin{array}{c}{\theta}_k\sim N\left(\mu,\:{\sigma}^2\right)\:(k=1,\dots,K)\end{array}$$

where ${\theta}_{k}$ is the treatment effect for indication $k$ on the scale of log odds, shrunk toward a common mean $\mu$ with between-group variance ${\sigma}^{2}$. A normal distribution with a large variance can be taken as the prior for $\mu$ to reflect the ignorance on the mean treatment effect across all indications. The variance parameter $\:{\sigma\:}^{2}$ controls the degree of information borrowing. A small value of ${\sigma}^{2}$ induces strong borrowing, while a large ${\sigma}^{2}$ induces little borrowing. In the extreme case where ${\sigma}^{2}=0$, the BHM is identical to pooled analysis; while if ${\sigma}^{2}\to\infty$, the BHM degenerates to independent analysis. Usually, ${\sigma\:}^{2}$ is estimated jointly with other parameters and a prior distribution is assigned to ${\sigma\:}^{2}$ at the design stage, e.g., an inverse gamma distribution $IG(a,b)$ with shape parameter $\:a$ and scale parameter $b$. 

During trial design, prior ESS can be assessed under possible scenarios to help determine whether the strength of borrowing is appropriate. When MSE of the estimator of $\:{p}_{k}$ is the target measure for deriving ESS, the posterior ESS is then the sample size required for an independent analysis to generate a comparable level of MSE with the Bayesian estimator, while the prior ESS is the posterior ESS minus the actual enrolled sample size. For indication $k$, the distance in MSE between independent and hierarchical analyses is defined as

3 $$\begin{aligned}&E\left[\left(\widehat p_k^I-p_k\right)^2\vert m_k+n_k,p_k\right]\\&-E\left[\left(\widehat p_k^B-p_k\right)^2\vert n_1,\dots,n_K,p_1,\dots,p_K\right] \end{aligned}$$

where ${\widehat{p}}_{k}^{I}$ is the maximum likelihood estimate (MLE) under independent analysis with a sample size of $\:{m}_{k}+{n}_{k}$, and ${\widehat{p}}_{k}^{B}$ is the estimate derived by BHM (the posterior mean serves as the point estimator for Bayesian approach hereinafter). The sampling distribution of ${\widehat{p}}_{k}^{I}$ is analytically tractable as $\widehat p_k^I\times\left(m_k+n_k\right)\sim\mathrm{Bin}(m_k+n_k,p_k)$. While for ${\widehat{p}}_{k}^{B}$, the sampling distribution can be obtained empirically by Monte Carlo simulation, thus facilitating the search for $\:{m}_{k}$ that minimizes (3). The MSE-based ESS inherently accounts for both the bias and variance components of the estimators. For instance, the MSE of ${\widehat{p}}_{k}^{B}$ can be decomposed into its variance plus the square of its bias as $\mathbb{E}\left[{\left({\widehat{p}}_{k}^{B}-{p}_{k}\right)}^{2}\right]=Var\left({\widehat{p}}_{k}^{B}\right)+{\left[Bias\right({\widehat{p}}_{k}^{B}\left)\right]}^{2}$. In cases of substantial bias that contributes to the increase of MSE, the prior ESS may be negative, indicating strong heterogeneity across indications.

At the analysis stage, the true value of ${p}_{k}$ is unknown and can be replaced by the MLE $\:{\widehat{p}}_{k}={y}_{k}/{n}_{k}$ when deriving the ESS, where $\:{y}_{k}$ denote the observed number of responders with indication $\:k$. Then, the distance in MSE becomes

4 $$\begin{aligned} & {\mathbb{E}}_{{Y}_{k}^{{m}_{k}+{n}_{k}}}\left[{\left({\widehat{p}}_{k}^{I}-{\widehat{p}}_{k}\right)}^{2}|{m}_{k}+{n}_{k}\right]\\ & -{\mathbb{E}}_{{Y}_{k}^{{n}_{k}}}\left[{\left({\widehat{p}}_{k}^{B}-{\widehat{p}}_{k}\right)}^{2}|{n}_{1},\dots,{n}_{K},{y}_{-k}\right].\end{aligned}$$

where ${\widehat{p}}_{k}^{I}={y}_{k}^{{m}_{k}+{n}_{k}}/({m}_{k}+{n}_{k})$ and ${\widehat{p}}_{k}^{B}$ is the estimate obtained by fitting the Bayesian model conditional on $\:{y}_{k}^{{n}_{k}}$ and data from indications other than $\:k$ (i.e., ${y}_{-k}$). Here, $\:{y}_{k}^{{m}_{k}+{n}_{k}}$ (or $\:{y}_{k}^{{n}_{k}}$) denotes the specific value of the random variable ${Y}_{k}^{{m}_{k}+{n}_{k}}$ (or $\:{Y}_{k}^{{n}_{k}}$), which is assumed to follow a binomial distribution with probability ${\widehat{p}}_{k}$ and size of $\:{m}_{k}+{n}_{k}$ (or $\:{n}_{k}$). The MSE is then computed by further averaging on the distribution of $\:{Y}_{k}^{{m}_{k}+{n}_{k}}$ (or $\:{Y}_{k}^{{n}_{k}}$). 

An alternative modelling strategy is to borrow information on the treatment difference compared to a reference, that is, ${\theta}_{k}=\text{l}\text{o}\text{g}\text{i}\text{t}\left({p}_{k}\right)-\text{l}\text{o}\text{g}\text{i}\text{t}\left({p}_{k0}\right)$, where $\:{p}_{k0}$ denotes the response rate of the standard of care (SoC) for indication $\:k$. Quantifying ESS follows analogous procedures with the above framework.

### ESS-based designs

The ESS can serve as a basis for some more complex basket trial designs, and here three extensions are given.

#### Model selection based on ESS

It may be reasonable to utilize independent analysis if there are tumor types with negative prior ESSs at the analysis stage, as there may be substantial bias and no benefit for reducing MSE. When the number of tumor types is large while only few tumor types have negative prior ESSs, it may also be reasonable to exclude those tumor types (often with very high or very low response rates) from the hierarchical model.

#### ESS-based CBHM

The application of traditional BHM suffers from inaccuracy in estimating $\:{\sigma\:}^{2}$, especially when $\:K$ is small. To deal with this issue, the calibrated BHM (CBHM) proposed by Chu and Yuan (2018) [11] takes the strategy of empirical Bayes to determine the value of ${\sigma}^{2}$ as

5 $$\begin{array}{c}{\sigma}^2=g\left(T\right)=\text{exp}\left\{a+b\times\text{log}\left(T\right)\right\}\end{array}$$

where $\:T$ is a measure for between-group heterogeneity, e.g., the chi-square statistic. Under specific scenarios, $\:a$ and $\:b$ are calibrated to achieve a desired level of ${\sigma\:}^{2}$. In the original paper of CBHM, the target ${\sigma\:}^{2}$ is set as 1 under the global alternative scenario where the investigational therapy is efficacious for all indications; while under the mixed scenario where the therapy demonstrates efficacy in only one indication, the desired $\:{\sigma\:}^{2}$ is set as 80. It is therefore natural to consider using prior ESS to assist in calibrating $\:a$ and $\:b$, which is more interpretable and transparent than target ${\sigma\:}^{2}$. 

#### ESS-based power prior

Baumann et al. proposed to apply power prior (PP) in Bayesian basket trials [39], extended from Fujikawa et al. (2020) [40]. Under this strategy, the amount of information that is shared between two baskets is only determined by their pairwise similarity, therefore avoiding fitting computation-expensive BHM. Specifically, the posterior distribution of $\:{p}_{k}$ has the closed form

6 $$\begin{array}{c}Beta\left(s_{1,k}+\sum_{i=1}^Kw_{k,i}\times\:y_i,s_{2,k}+\sum_{i=1}^Kw_{k,i}\times\left({n_i-y}_i\right)\right)\end{array}$$

where $\:{w}_{k,i}$ is the borrowing weight of indication $\:i$ that is used for the analysis of indication $k$ with the special case of $\:{w}_{k,k}=1$. ${s}_{1,k}$ and $\:{s}_{2,k}$ are parameters of the initial Beta prior. Weights $\:{w}_{k,i}$ can be given by

7 $$\begin{array}{c}w_{k,i}=\frac1{1+\text{exp}\left\{a+b\times\text{log}\left(S_{k,i}\right)\right\}},\end{array}$$

where $a$ and $\:b$ are tuning parameters that needs to be calibrated. ${S}_{k,i}={\text{m}\text{a}\text{x}({n}_{k},{n}_{i})}^{1/4}{S}_{KS;k,i}$ where ${S}_{KS;k,i}$ is the Kolmogorov-Smirnov (KS) statistic between indication $\:k$ and $i$. Similar to ESS-based CBHM, prior ESS can also serve as the reference when calibrating $\:a$ and $\:b$ for the PP approach. 

The posterior distribution of $\:{p}_{k}$ (6) can be interpreted as borrowing an additional sample with size of $\sum_{i=1}^{K}{w}_{k,i}\times{n}_{i}-{n}_{k}$ from other indications. Nevertheless, to ensure comparability among all methods and to quantitatively assess the impact of Bayesian borrowing on the MSE of estimation, in this paper we employ the MSE-based ESS for this design.

### Numerical study

The primary results of the RAGNAR study, a single-arm phase 2 basket trial designed to evaluate the safety and activity of erdafitinib in previously treated patients with FGFR-altered advanced solid tumors [14], are reanalyzed to illustrate how to assess the degree of information borrowing by prior ESS (R code is given in the Additional file 1). The primary endpoint is the objective response rate (ORR) based on RECIST v1.1 (for non-CNS tumors) or RANO (for CNS tumors). In the RAGNAR study, the same BHM as model (2) is applied where the prior distributions are $\mu\sim N(-2.19,2^2)$ and ${\sigma}^2\sim\mathrm{IG}\left(\text{0.375,1.5}\right)$. As mentioned by the statistical analysis plan (SAP) of the RAGNAR study, such priors ensure certain degree of borrowing (the readers may refer to the supplementary appendix of the published paper [14] for more details). We also calculate prior ESS when the prior of $\:{\sigma\:}^{2}$ is $\:IG(0.0005,0.000005)$, which reflects a small amount of heterogeneity across indications [6]. “Prior A” and “Prior B” are denoted as these two prior settings.

We further demonstrate how prior ESS can be applied to supplement operating characteristics during the design stage. Consider a basket trial where a total of four tumor types are under investigation and the primary endpoint is ORR. The sample size for each tumor type is 30, and the same Bayesian models with settings of Prior A and Prior B are used. Five scenarios, where the true ORRs may vary across tumor types, are included in the simulation study. Assuming a null ORR (ORR of the the SoC) of 0.15 and a lowest clinically meaningful ORR of 0.3 for each tumor type, Scenarios 1 and 2 are global null and global alternative scenarios, respectively. In Scenarios 3–5, the treatment effects are heterogeneous across tumor types, where the potential risks of borrowing need to be carefully evaluated. The hypothetical trial is simulated 10,000 times for each scenario to derive the MSE and prior ESS. We also evaluate the indication-specific type I error rate and power, the most common frequentist operating characteristics in published basket trial designs. For each indication $k$, we consider the null hypothesis ${H}_{0k}:{p}_{k}\le0.15$ against the alternative hypothesis ${H}_{1k}:{p}_{k}>0.15$. The indication-specific type I error rate and power are evaluated by the probabilities of rejecting the null hypothesis for tumor types with an ORR of 0.15 and 0.3, respectively. If the posterior probability $\text{Pr}\left({p}_{k}>0.15|Data\right)>{\phi}_{k}$, the null hypothesis will be rejected. To make the two prior settings comparable, ${\phi}_{k}$ are the same for all indications and are calibrated to control the indication-specific type I error rate under Scenario 1 around 5%.

Three ESS-based borrowing strategies introduced before are also compared. As for model selection based on ESS (denoted as “ESS-based MS” hereinafter), Prior B is used for fitting BHM and deriving ESS, as negative prior ESSs occur only in a small number of simulated trials when applying Prior A and model selection has negligible effects on the operating characteristics. If there are any tumor types with negative prior ESSs at the analysis stage, MS takes the strategy of independent analysis where a vague prior $Beta\left(\text{0.5,0.5}\right)$ is used. In terms of ESS-based CBHM, the tuning parameters $\:a$ and $\:b$ in Eq. (5) are calibrated by grid search and the target is that the prior ESS for each arm in Scenario 2 is around 30 (i.e., the same as ${n}_{k}$) while the prior ESS for tumor type 4 in Scenario 5 is 0, resulting in $\:a=-2$ and $b=0.4$. Similarly, for ESS-based PP, tuning parameters $a$ and $\:b$ in Eq. (7) are calibrated to 2 and 0.4, with the same target as ESS-based CBHM. 

## Results

### Reanalysis of the RAGNAR study

**Fig. 1:**
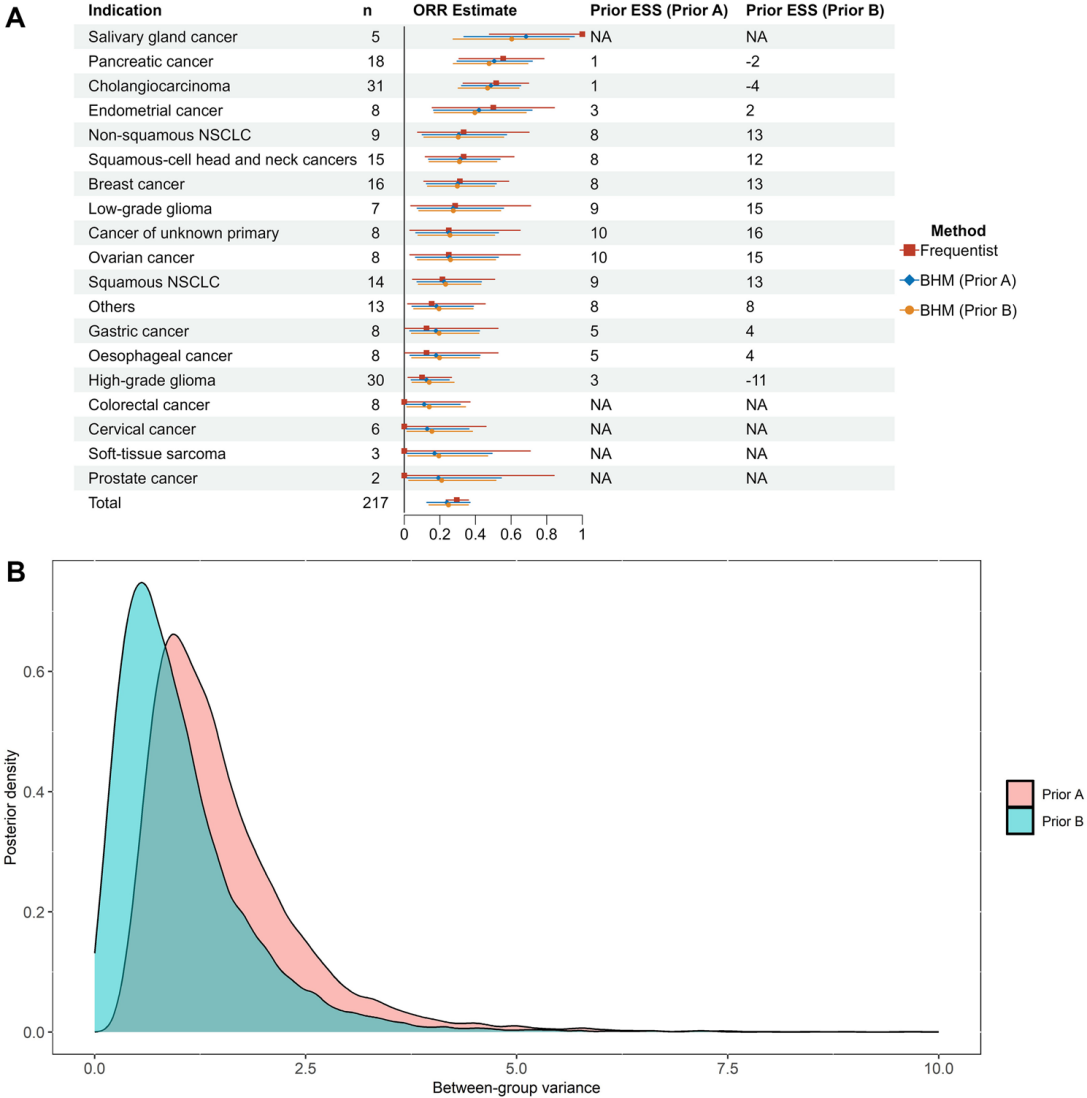
Frequentist and Bayesian estimates of ORR across tumor types in the RAGNAR study. **A** ORR estimates and prior ESS. **B** Posterior distributions of the between-group variance. NA = Not available. The frequentist estimate is the observed ORR with a 95% confidence interval based on Clopper and Pearson method. The BHM estimate is the posterior mean with a 95% equal-tailed credible interval

The frequentist and Bayesian estimates of ORR, as well as prior ESS for each tumor type are shown in Fig. 1A. When no information is shared across tumor types, the uncertainties of frequentist estimates are quite large, manifested by the wide 95% confidence intervals (CI). The estimates produced by BHM, on the other hand, differ from the frequentist estimates in two aspects: (1) the indication-specific treatment effects are shrunk toward a common mean; (2) the variances of estimates are smaller for most indications. Taking the breast cancer as an example, the frequentist estimate is 31.3% with a 95% CI (11.0%, 58.7%), while the Bayesian estimate under Prior A is 30.0% with a 95% equal-tailed credible interval (CrI) (12.5%, 51.7%), more close to the pooled ORR across all indications (29.5%). In this case, there is a desired bias-variance tradeoff, as the prior ESS is positive, suggesting the application of BHM has a benefit in reducing MSE. It is also found that for tumor types with ORR close to the pooled ORR, the prior ESSs are generally larger. For tumor types with ORR far away from the pooled ORR, the prior ESSs are small or even become negative. In such cases, Bayesian estimates are likely to be biased, so as to offset the benefit in reducing variance. It is then recommended to carefully consider the rationale of including such indications in the Bayesian model. It is also worth noting that the prior ESS is not available under extreme cases where the observed ORR is 0% or 100%. This is due to the fact that the variance for a binary variable is 0 if the probability parameter is 0% or 100%, and the MSE in terms of independent analysis would be always 0, making the derivation of ESS impossible. The inability to determine ESS in this case is not a serious issue in our perspective, because sponsors will definitely continue clinical developments or seek regulatory approvals for tumor types with an observed ORR of 100% if there is no particular concern about safety. While for tumor types with an observed ORR of 0% and small sample sizes, it is far from sufficient to support drug efficacy based on Bayesian estimates (e.g., around 20% for prostate cancer), and more clinical evidence is required.

In terms of the comparison between Prior A and Prior B, it is noticeable that, under Prior B, the amounts of information borrowed for some indications are huge, and the prior ESSs far exceed the study sample sizes. For example, the enrolled sample size for low-grade glioma is 7, while the prior ESS under Prior A and Prior B are 9 and 15, respectively. For tumor types with high observed ORR (e.g., cholangiocarcinoma) or low observed ORR (e.g., high-grade glioma), Prior B may induce negative prior ESSs, suggesting that Prior B results in a stronger borrowing and larger biases. This conclusion can be confirmed by the posterior distributions of ${\sigma}^{2}$ (Fig. 1B), where Prior B produces a smaller variance. Generally speaking, prior ESS is a much more interpretable metric to quantify the degree of borrowing than the between-group variance.

### Evaluating prior ESS during trial design

The simulation results are summarized in Table 1, where the prior ESS retains one decimal place by linear interpolation between two integers that minimize the absolute value of (3). Most of the MSE-based prior ESSs are positive (even in heterogeneous scenarios), indicating that applying BHM in such a context is beneficial in reducing MSE. As anticipated, the MSE is larger in heterogeneous scenarios, and as a result, the indication-specific prior ESSs are smaller. For example, under Prior B, the MSE-based prior ESS for tumor type 1 is 73.7 and − 7.7 in Scenarios 1 and 3, respectively (the MSE is 1.2 × 10^− 3^ and 9.4 × 10^− 3^ for these two prior settings). There is also a higher probability of deriving a negative prior ESS in heterogeneous scenarios, as the BHM would lead to biased estimates, which in turn increases MSE.

**Table 1:** Prior ESS and operating characteristics under five scenarios for BHM with two prior settings

Tumor type	ORR	Prior A				Prior B			
		MSE-based Prior ESS	VR-based Prior ESS	MSE ×10^− 3^	Reject %	MSE-based Prior ESS	VR-based Prior ESS	MSE ×10^− 3^	Reject %
Scenario 1									
1	0.15	10.6	10.6	3.1	**5.0**	73.7	73.7	1.2	**4.8**
2	0.15	10.0	10.0	3.2	**5.1**	72.9	72.8	1.2	**5.2**
3	0.15	11.0	11.0	3.1	**5.2**	73.4	73.4	1.2	**5.0**
4	0.15	11.7	11.7	3.1	**4.8**	75.1	75.1	1.2	**5.0**
Scenario 2									
1	0.3	7.4	7.5	5.6	72.7	72.8	73.0	2.0	96.3
2	0.3	6.7	6.7	5.7	71.2	71.0	71.2	2.1	96.2
3	0.3	7.7	7.7	5.6	72.5	72.6	72.8	2.0	96.1
4	0.3	8.3	8.3	5.5	72.4	74.1	74.3	2.0	96.7
Scenario 3									
1	0.3	4.0	5.6	6.2	64.8	−7.7	18.7	9.4	46.8
2	0.15	8.8	8.9	3.3	**6.5**	26.5	42.7	2.3	**16.2**
3	0.15	9.6	9.8	3.2	**6.4**	26.6	43.3	2.3	**16.1**
4	0.15	10.2	10.4	3.2	**6.4**	28.0	45.4	2.2	**15.9**
Scenario 4									
1	0.3	5.3	6.1	6.0	71.1	6.3	29.3	5.8	74.6
2	0.3	4.5	5.4	6.1	70.3	6.0	28.5	5.8	74.0
3	0.15	8.1	9.0	3.3	**6.7**	−0.3	25.9	4.3	**31.4**
4	0.15	8.7	9.6	3.3	**6.7**	0.2	27.1	4.2	**30.9**
Scenario 5									
1	0.3	6.4	6.7	5.8	72.5	31.4	48.2	3.4	88.7
2	0.3	5.6	6.0	5.9	71.1	30.4	46.9	3.5	88.4
3	0.3	6.6	6.9	5.7	72.4	31.6	48.6	3.4	89.1
4	0.15	6.9	9.0	3.5	**6.8**	−14.6	17.2	8.3	**50.5**

Reject = probability of rejecting the null hypothesis, where texts in bold indicate type I error rates and others indicate power

Compared to Prior A, the degree of borrowing in terms of Prior B is much higher, consistent with the findings of the reanalysis of the RAGNAR study. In Scenarios 1 and 2, the prior ESS for each tumor type is around 70 under Prior B, i.e., more than two times the study sample size. In Scenario 5, the prior ESS is −14.6 for tumor type 4 under Prior B, while is still positive under Prior A. The prior ESSs, not surprisingly, are consistent with the empirical type I error rate and power. Specifically, Prior B induces a strong borrowing and thus a much higher power in Scenario 2. However, it also results in large bias and serious type I error inflation (e.g., 50.5% for tumor type 4 in Scenario 5). On the other hand, the power under Prior A for each tumor type is around 70%, not much of an obvious improvement from 58% in an independent analysis with an one-sided significance level of 5%, but there is only a slight inflation of type I error and may be more acceptable in some cases. Additional sensitivity analyses are also conducted to evaluate the impact of the prior distribution of $\:{\sigma\:}^{2}$ on prior ESS (See the Additional file 1 for detailed results).

The ESS based on variance ratio (VR) is also compared with the MSE-based ESS (Table 1). Under Prior A and under homogeneous scenarios (Scenarios 1 and 2) with Prior B, the two types of prior ESS demonstrate comparable results. However, in heterogeneous scenarios accompanied by strong borrowing (Scenarios 3–5 under Prior B), the VR-based prior ESS appears less sensitive. Specifically, the VR-based prior ESS exhibits a narrower range of variation across scenarios, and no negative prior ESS values are observed. This discrepancy arises because the VR-based approach primarily captures changes in variance and does not directly incorporate the magnitude of estimation bias. Consequently, it fails to adequately reflect the potential drawbacks of borrowing, i.e., substantial bias in estimates. It is then recommended to use the MSE-based prior ESS to quantify the degree of borrowing in the context of Bayesian basket trial designs. For further details on the calculation of VR-based prior ESS and its results in the reanalysis of the RAGNAR study, refer to the Additional file 1.

### Comparisons of ESS-based borrowing strategies

**Fig. 2:**
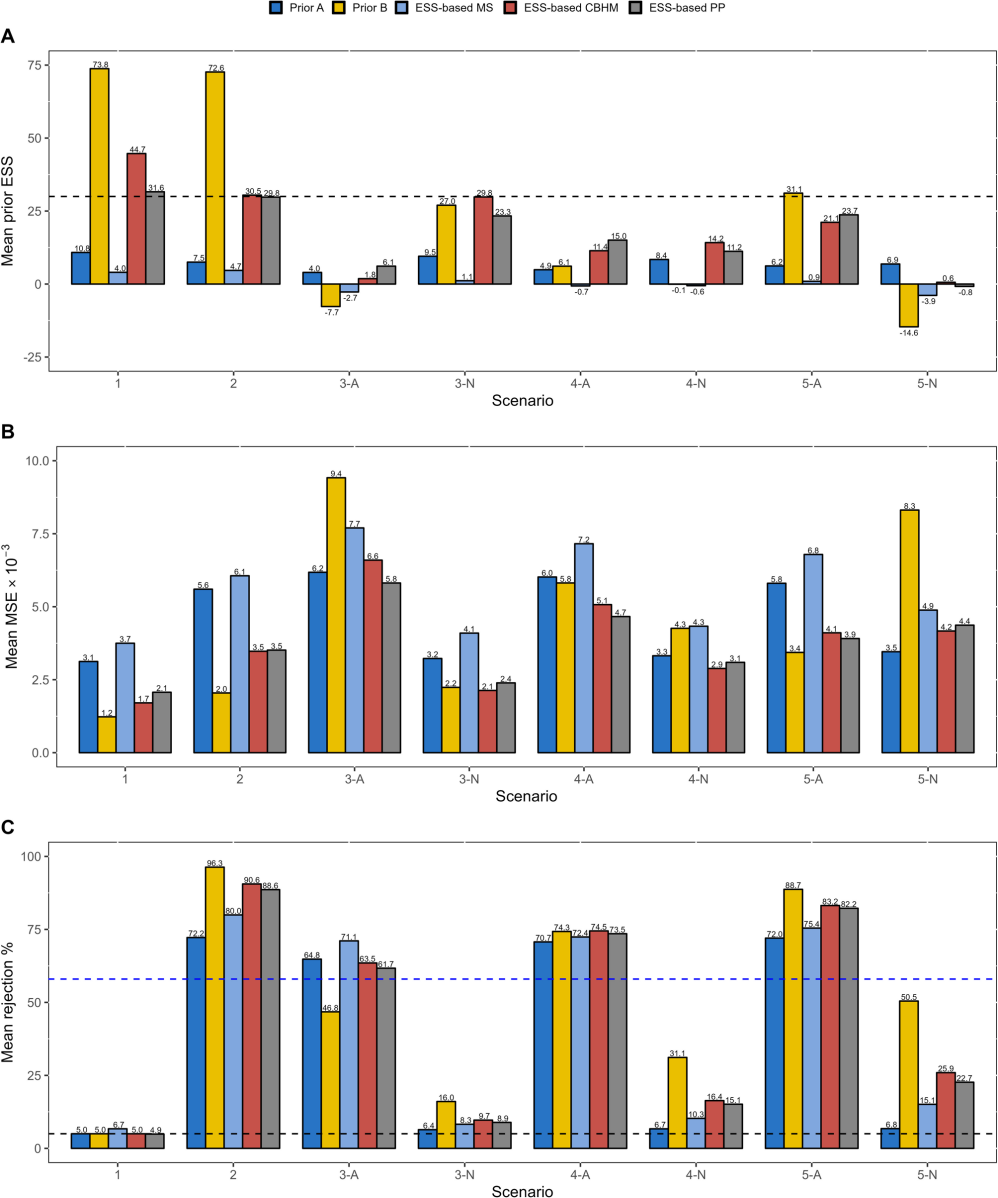
Comparison of ESS-based borrowing strategies on (**A**) prior ESS, (**B**) MSE, and (**C**) probabilities of rejecting the null hypothesis. Notes: Horizontal axis labels “A” and “N” denote the alternative and null study arms, respectively. The dashed line in panel A indicates the level at which the prior ESS is equal to 30. The dashed lines in panel C correspond to the nominal significance level (5%) and target power threshold (58%)

The comparisons of three ESS-based borrowing strategies are summarized in Fig. 2, where metrics of study arms with the same value of ORR are averaged. The independent analysis is selected in most simulated trials when using ESS-based MS, resulting in statistical performance similar to that of BHM with Prior (A) The most critical issue of ESS-based MS is that it is not flexible in adjusting the degree of information borrowing to reach a desired level during trial design once the BHM and method for ESS calculation are fixed. On the contrary, ESS-based CBHM and ESS-based PP have distinct objectives when calibrating design parameters and can be adjusted according to specific clinical backgrounds. In our simulation, the prior ESSs of arms in Scenario 2 and study arm with no treatment effect in Scenario 5 are well controlled as we anticipated. It is also worth noting that the prior ESSs these two strategies produce are not negatives with large absolute values, indicating benefits in bias-variance tradeoff. Since the calibration objective is the same, the statistical performance of the two strategies is also similar. They can achieve around 90% power in the global alternative scenario, close to the 96.3% by BHM with Prior B, but the worst indication-specific type I error rate is only half of that by BHM with Prior (B) Certainly, it cannot be denied that it is impossible to control the type I error rate at 0.05 for all null study arms in those mixed scenarios (Scenarios 3–5), otherwise there will be no power gain. But in general, adjusting the degree of information borrowing through prior ESS can make Bayesian models more interpretable and transparent.

In this simulation study, it is challenging to employ VR-based prior ESS as the calculation method for ESS-based MS, CBHM, and PP. As demonstrated in Table 1, VR-based prior ESS is not sensitive enough to heterogeneous scenarios. Only when there is a strong borrowing (Prior B in Table 1) would there be apparent differences in prior ESS across indications. Consequently, calibrating design parameters to the same target (the prior ESS for each arm in Scenario 2 is around 30 while the prior ESS for tumor type 4 in Scenario 5 is 0) as MSE-based approach becomes infeasible. Furthermore, as negative prior ESS values rarely occur, the ESS-based MS is ineffective under these conditions.

## Discussion

Using Bayesian approaches to borrow information in basket trials is receiving increasing attention, but there is currently no recognized solution for quantifying the degree of borrowing. In this paper, we apply the concept of ESS to Bayesian basket trials and demonstrate its great interpretability at the analysis and design stages separately. As concluded by Broglio et al. [41], increasing the complexity of the Bayesian model would not significantly improve the design performance when the sample size is not large (which is common in the context of basket trials), and the optimization of model parameters, such as the prior settings, deserves more attention. Therefore, this paper mainly presented cases of prior ESS under the traditional BHM. Naturally, as long as the MSE of the indication-specific estimator remains the primary concern, the ESS tools can be readily extended to other Bayesian models.

The prior ESS is a good reference for the interpretation of the Bayesian analysis results. For example, negative prior ESS values indicate that the bias introduced by information borrowing outweighs the benefit of variance reduction. A negative prior ESS can be interpreted as a quantitative warning against inappropriate borrowing across indications. Specifically, in the design stage, a negative prior ESS typically arises when the true treatment effect of a particular indication markedly deviates from the pooled effect across indications. At the analysis stage, a negative prior ESS often occurs when there is a considerable discrepancy between the results from independent analyses and those derived from Bayesian models. The use of priors that encourage aggressive borrowing can lead to negative prior ESSs even in moderately heterogeneous scenarios, as such priors may amplify the estimation bias.

By convention, prior information should not dominate Bayesian statistical inferences. In terms of basket trials that are concerned here, the prior ESS for a specific tumor type should not exceed the actual enrolled sample size. But in reality, this is something that needs to be discussed on a case-by-case basis. For example, in the context of rare tumors, a slightly larger prior ESS may be allowed. We do not attempt to discuss the rationale for such a convention in this paper, but rather recommend that sponsors and regulatory agencies should pay particular attention to this issue and communicate to reach a consensus on the model settings of the Bayesian methods to be used.

Future research directions may include comparing the characteristics of prior ESS when selecting different target measures in Eq. (1), investigating prior ESS for Bayesian models with multiple endpoints, and explicitly incorporating ESS into the decision-making framework. For randomized basket trials, we can further consider adjusting the allocation proportions adaptively between the experimental arm and the control arm. The relevant research is currently ongoing.

## Conclusions

It is crucial to quantify the degree of information borrowing by prior ESS in the context of Bayesian basket trials. This quantification not only assists trialists in reasonably evaluating and interpreting Bayesian analysis results but also aids in conducting sensitivity analyses. Consequently, it enables the determination of an appropriate amount of information to borrow across indications.

## Supplementary Information

Supplementary Material 1.

## Data Availability

The data involved in this paper are obtained from the publication of the RAGNAR study (DOI: 10.1016/S1470-2045(23)00275-9) or by simulation.

## Abbreviations

BHM: Bayesian hierarchical model
CBHM: Calibrated BHM
CI: Confidence intervals
CrI: Credible interval
ESS: Effective sample size
MLE: Maximum likelihood estimate
MSE: Mean squared error
NSCLC: Non–small-cell lung cancer
ORR: Overall response rate
POC: Proof-of-concept
PP: Power prior
SoC: Standard of care
VR: Variance ratio
