# Supplementary material for: Enhancing interpretability for Bayesian basket trial designs by effective sample size
Source: BMC Med Res Methodol. 2025 Dec 16;25:278. doi: 10.1186/s12874-025-02715-x (PMC12709804; doi:10.1186/s12874-025-02715-x)
Supplement: Supplementary file 1 — Supplementary Material 1. [file 12874_2025_2715_MOESM1_ESM.pdf]

Additional file 1

Supplemental material for 'Enhancing interpretability for Bayesian basket trial designs by effective sample size'

Examples of R code

```
## The following is R code for data analysis:
library(rjags)
library(readxl)
library(parallel)
data <- read_xlsx('Data example.xlsx') ## Input data
n <- data$n ## Sample size
y <- data$y ## Responders
K <- length(y) ## Number of tumor types
p_hat <- y/n ## Naive estimates of ORR
Indication <- data$Indication
mu0 <- -2.19 ## Prior mean of the average treatment effect (log odds scale)
var0 <- 4 ## Prior variance of the average treatment effect (log odds scale)
ig_a <- 0.375 ## Shape parameter of the inverse gamma distribution
ig_b <- 1.5 ## Scale parameter of the inverse gamma distribution
mfold <- 10 ## The upper limit for searching prior ESS is mflod times the sample size for each
indication

nchains <- 3 ## Number of MCMC chains
nadapt <- 1000 ## Number of iterations for adaptation
niter <- 5000 ## Number of iterations for sampling
nthin <- 1 ## Thinning interval
ncore <- 2 ## Number of cores used

## Loop for calculating the 95% CI of ORR, based on Clopper and Pearson method
CI_Lower <- rep(NA, length(n))
CI_Upper <- rep(NA, length(n))
for (i in 1:length(n)){
   fit <- binom.test(y[i],n[i])
   CI_Lower[i] <- fit$conf.int[1]
   CI_Upper[i] <- fit$conf.int[2]
}
temp <- binom.test(sum(y),sum(n)) ## Calculate the 95% CI of the pooled ORR
Indication <- c(data$Indication, 'Total')
p_hat <- c(p_hat, temp$estimate)
CI_Lower <- c(CI_Lower, temp$conf.int[1]); CI_Upper <- c(CI_Upper, temp$conf.int[2])
## Bayesian analysis ##
## Generate random initial values for parameters
```

```
jags.init <- list()
for (i in 1:nchains){
  jags.init[[i]] <- list(.RNG.name = 'base::Wichmann-Hill', .RNG.seed = i)
}
model <-
  'model{
        for (k in 1:K){
          y[k] ~ dbin(p[k], n[k])
          logit(p[k]) <- logit_p[k]
          logit_p[k] ~ dnorm(mu, tau)
        }
        mu ~ dnorm(mu0, tau0)
        tau ~ dgamma(a, b)

        p_mu <- exp(mu)/(1+exp(mu))
        variance <- 1/tau
   }'
jags.data <- list('y'=y,'n'=n,'K'=K,'mu0'=mu0,'tau0'=1/var0,
                  'a'=ig_a,'b'=ig_b)

jags.fit <- jags.model(file = textConnection(model), data = jags.data, inits = jags.init,
                       n.adapt = nadapt, n.chains = nchains, quiet = TRUE) ## Compile
model
output <- coda.samples(jags.fit, variable.names = c('p','p_mu','variance'), n.iter=niter,
                       thin = nthin, progress.bar = 'none') ## Generate posterior samples
temp <- summary(output) ## Obtain the posterior mean estimate and 95% CrI
parindex <- which(grepl('p', rownames(temp$statistics))) ## Target the parameters of interest
result <- data.frame(Indication=Indication, N=c(n, sum(n)), p_hat, CI_Lower, CI_Upper,
                     Postmean=temp$statistics[parindex,1],
                     CrI_Lower=temp$quantiles[parindex,1],
                     CrI_Upper=temp$quantiles[parindex,5])

ESS_MSE <- rep(NA, K)
ESS_Var <- rep(NA, K)

Modelfit <- function(ypotential, n, y, K, k, nchains, nadapt, niter, nthin, mu0, var0, ig_a, ig_b){
  library(rjags)
  y_new <- y
  y_new[k] <- ypotential ## potential outcome
  ## Generate random initial values for parameters
  jags.init <- list()
  for (i in 1:nchains){
    jags.init[[i]] <- list(.RNG.name = 'base::Wichmann-Hill', .RNG.seed = i)
  }
```

```
  model <-
    'model{
      for (k in 1:K){
        y[k] ~ dbin(p[k], n[k])
        logit(p[k]) <- logit_p[k]
        logit_p[k] ~ dnorm(mu, tau)
      }
      mu ~ dnorm(mu0, tau0)
      tau ~ dgamma(a, b)

      p_mu <- exp(mu)/(1+exp(mu))
      variance <- 1/tau
    }'
  jags.data <- list('y'=y_new,'n'=n,'K'=K,'mu0'=mu0,'tau0'=1/var0,
                    'a'=ig_a,'b'=ig_b)
  jags.fit <- jags.model(file = textConnection(model), data = jags.data, inits = jags.init,
                         n.adapt=nadapt, n.chains=nchains, quiet=TRUE) ## Compile model
  output <- coda.samples(jags.fit, variable.names = c('p'), n.iter=niter,
                         thin = nthin, progress.bar = 'none') ## Generate posterior samples
  return(list(postmean=summary(output)$statistics[k,1])) ## Return the posterior mean estimate
}

for (k in 1:K){
  pk_hat <- p_hat[k]
  ## Posterior variance and posterior mean under BHM
  Var_borrow <- summary(output)$statistics[which(rownames(summary(output)$statistics) == paste0('p[',k,']')),2]^2
  Mean_borrow <- result$Postmean[which(rownames(result) == paste0('p[',k,']'))]
  ## Variance of pk_hat (the estimator under the independent analysis)
  Var_noborrow <- pk_hat*(1-pk_hat)/n[k]
  if (pk_hat == 0 | pk_hat == 1){
    ESS_MSE[k] <- 'NA'
    ESS_Var[k] <- 'NA'
    next
  }
  ## ESS based on variance ratio
  ESS_Var[k] <- round((Var_noborrow/Var_borrow - 1)*n[k],1)

  ## MSE under BHM
  cl <- makeCluster(mc <- getOption('cl.cores', ncore))
  simVector <- 0:n[k]
  temp <- clusterApplyLB(cl, simVector, Modelfit, n, y, K, k, nchains, nadapt, niter, nthin,
```

```
                                    mu0, var0, ig_a, ig_b)
  stopCluster(cl)
  pk_hat_new <- sapply(temp[1:length(temp)], function(x) x$postmean)
  p_binom <- dbinom(0:n[k], n[k], pk_hat)
  MSE_borrow <- sum((pk_hat_new-pk_hat)^2*p_binom)
  ## MSE under independent analysis
  m <- seq(-n[k]+1, mfold*n[k])   ## The range for searching the prior ESS
  MSE <- rep(NA, length(m))
  for (j in 1:length(m)){
    p_binom <- dbinom((0:(m[j]+n[k])), m[j]+n[k], pk_hat)
    MSE[j] <- sum(((0:(m[j]+n[k]))/(m[j]+n[k]) - pk_hat)^2*p_binom)
  }
  ## ESS based on matching MSE
  ESS_MSE[k] <- m[which((abs(MSE-MSE_borrow)) == min(abs(MSE-MSE_borrow)))]
}
result$ESS_MSE <- c(ESS_MSE,'')
result$ESS_Var <- c(ESS_Var,'')
```

Data example:

Indication	n	y	p0
Salivary gland cancer	5	5	0.5
Pancreatic cancer	18	10	0.5
Cholangiocarcinoma	31	16	0.5
Endometrial cancer	8	4	0.3
Non-squamous NSCLC	9	3	0.3
Squamous-cell head and neck cancers	15	5	0.3
Breast cancer	16	5	0.3
Low-grade glioma	7	2	0.3
Cancer of unknown primary	8	2	0.3
Ovarian cancer	8	2	0.3
Squamous NSCLC	14	3	0.3
Others	13	2	0.2
Gastric cancer	8	1	0.2
Oesophageal cancer	8	1	0.2
High-grade glioma	30	3	0.2
Colorectal cancer	8	0	0.2
Cervical cancer	6	0	0.2
Soft-tissue sarcoma	3	0	0.2
Prostate cancer	2	0	0.5

The calculation of VR-based ESS

At the design stage, the prior ESS based on variance ratio (VR) is defined as

$$n_k\left(\frac{Var(\hat{p}_k^I|n_k,p_k)}{Var(\hat{p}_k^B|n_1,\dots,n_K,p_1,\dots,p_K)}-1\right), \tag{1}$$

where the primary concern is the reduction of variance on the estimator. Here $Var(\hat{p}_k^I|n_k,p_k)$ is obtained by $p_k(1-p_k)/n_k$ as $\hat{p}_k^I\times n_k \sim Bin(n_k,p_k)$, and $Var(\hat{p}_k^B|n_1,\dots,n_K,p_1,\dots,p_K)$ is derived by simulation. At the analysis stage, VR-based prior ESS becomes

$$n_k\left(\frac{Var(\hat{p}_k^I|n_k,y_k)}{Var(\hat{p}_k^B|n_1,\dots,n_K,y_1,\dots,y_K)}\right). \tag{2}$$

$Var(\hat{p}_k^I|n_k,y_k)$ is obtained by $\hat{p}_k(1-\hat{p}_k)/n_k$ according to asymptotics where $\hat{p}_k=y_k/n_k$ is the MLE, and $Var(\hat{p}_k^B|n_1,\dots,n_K,y_1,\dots,y_K)$ is the posterior variance of p_k when fitting Bayesian model.

Comparison between MSE-based and VR-based ESS for the reanalysis of the RAGNAR study

We calculate two types of ESS in the reanalysis of the RAGNAR study,and the comparative results are shown in Table S1. Similar to the simulation results shown in the main text, VR-based prior ESS may not be sensitive enough to the potential heterogeneity across indications. Specifically, the ranges of MSE-based prior ESS and VR-based prior ESS are 1~10 and -0.5~5.5 under Prior A. While under Prior B, these two ranges are -11~16 and -5.2~8.4 respectively.

Table S1. Comparison between MSE-based and VR-based ESS when reanalyzing the RAGNAR study.

Indication	N	ORR %	Prior A		Prior B	
			MSE-based Prior ESS	VR-based Prior ESS	MSE-based Prior ESS	VR-based Prior ESS
Salivary gland cancer	5	100	NA	NA	NA	NA
Pancreatic cancer	18	55.6	1	2.8	-2	2.4
Cholangiocarcinoma	31	51.6	1	3.3	-4	1.4
Endometrial cancer	8	50.0	3	4.1	2	6.1
Non-squamous NSCLC	9	33.3	8	5.4	13	7.6
Squamous-cell head and neck cancers	15	33.3	8	4.9	12	8.4
Breast cancer	16	31.3	8	5.3	13	7.6
Low-grade glioma	7	28.6	9	5.5	15	7.6
Cancer of unknown primary	8	25.0	10	5.1	16	7.4
Ovarian cancer	8	25.0	10	4.5	15	7.1
Squamous NSCLC	14	21.4	9	5.2	13	6.6
Others	13	15.4	8	3.4	8	4.3
Gastric cancer	8	12.5	5	2.3	4	3
Oesophageal cancer	8	12.5	5	2.2	4	3
High-grade glioma	30	10.0	3	-0.5	-11	-5.2
Colorectal cancer	8	0	NA	NA	NA	NA
Cervical cancer	6	0	NA	NA	NA	NA
Soft-tissue sarcoma	3	0	NA	NA	NA	NA
Prostate cancer	2	0	NA	NA	NA	NA
Total	217	29.5				

NA=Not available.

Sensitivity analysis on prior distributions of the between group variance

Suppose the desired ORR is 0.3, a sample size of 36 for a specific indication can result in an asymptotic variance 0.0058, corresponding to a confidence interval with a width of 0.3. If the desired ratio of information borrowed from other indications to the information of that indication is 20%, it is natural to set a sample size of 30 and calibrate parameters in order that the prior ESS is around 6; if the desired ratio of borrowing is 100%, the sample size for each indication is 18 and the prior ESS should be around 18. Based on the simulation setup in the main paper, here we investigate how the prior ESS and MSE vary with the prior distribution of σ^2. The inverse gamma prior $IG(a,b)$ is reparameterized with a prior mean μ_{σ^2} and a weight parameter w_{σ^2}, where $a = w_{\sigma^2}/2$ and $b = \mu_{\sigma^2}^2 w_{\sigma^2}/2$, eg, $IG(0.375,1.5)$ has a mean of 2 and a weight of 0.75. Fixing the prior mean at 2, we plot the variation of indication-specific prior ESS and MSE with the weight parameter when the sample size of each arm is 30 (Figure S1) and 18 (Figure S2). It can be seen that the larger MSE of the estimator, the smaller the prior ESS, indicating less borrowing of information across indications. It is generally recommended to evaluate the appropriateness of the prior distribution by ESS. Specifically, if the sample size is 30 per arm and the desired ratio of borrowing is 20%, a weight parameter of 1.2 is appropriate; while if the sample size is 18 per arm and the desired ratio of borrowing is 100%, a weight parameter of 0.05 is appropriate.

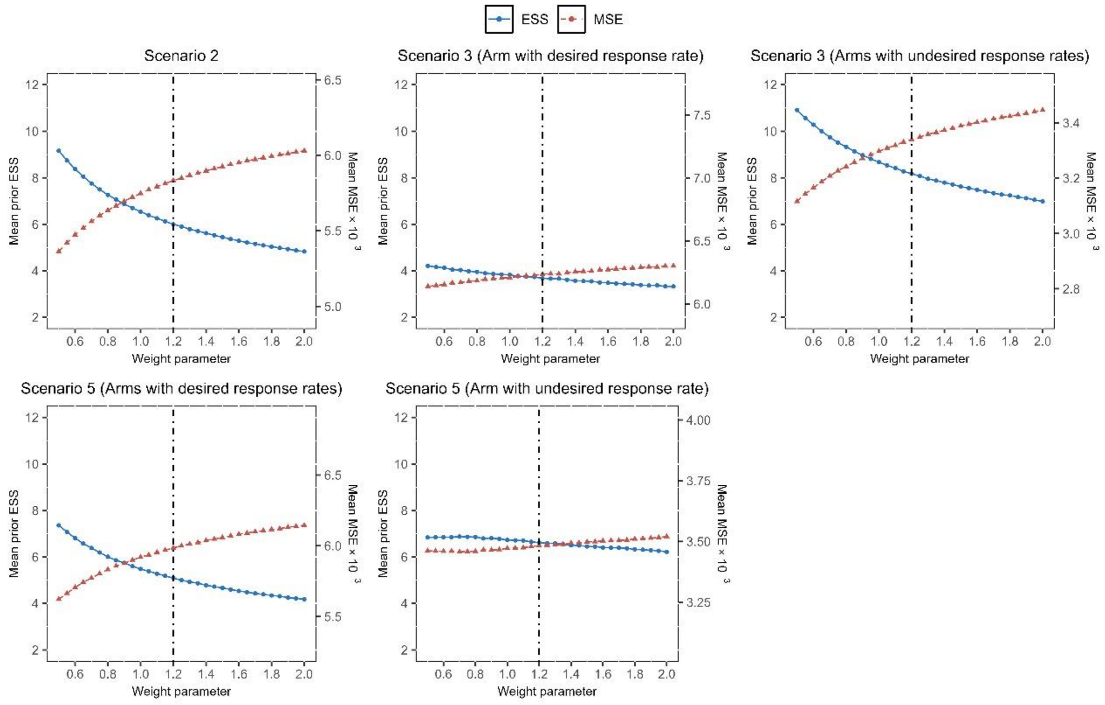

Fig. S1 Variation of indication-specific prior ESS and MSE with the weight parameter of the prior for σ^2 across multiple scenarios when the sample size of each arm is 30.

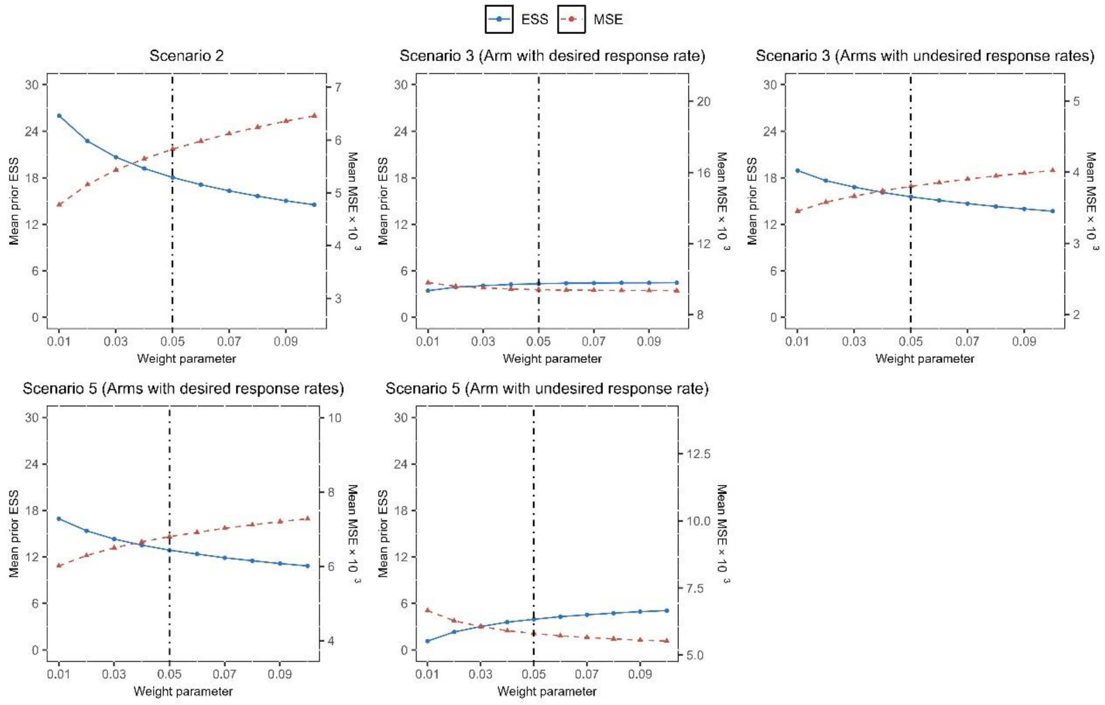

Fig. S2 Variation of indication-specific prior ESS and MSE with the weight parameter of the prior for σ^2 across multiple scenarios when the sample size of each arm is 18.
